# Supplementary material for: Combining Recombinase-Mediated Cassette Exchange Strategy with Quantitative Proteomic and Phosphoproteomic Analyses to Inspect Intracellular Functions of the Tumor Suppressor Galectin-4 in Colorectal Cancer Cells
Source: Int J Mol Sci. 2022 Jun 8;23(12):6414. doi: 10.3390/ijms23126414 (PMC9223697; doi:10.3390/ijms23126414)
Supplement: Supplementary file 1 [file ijms-23-06414-s001.zip › Figure S1.pdf]

Figure S1.

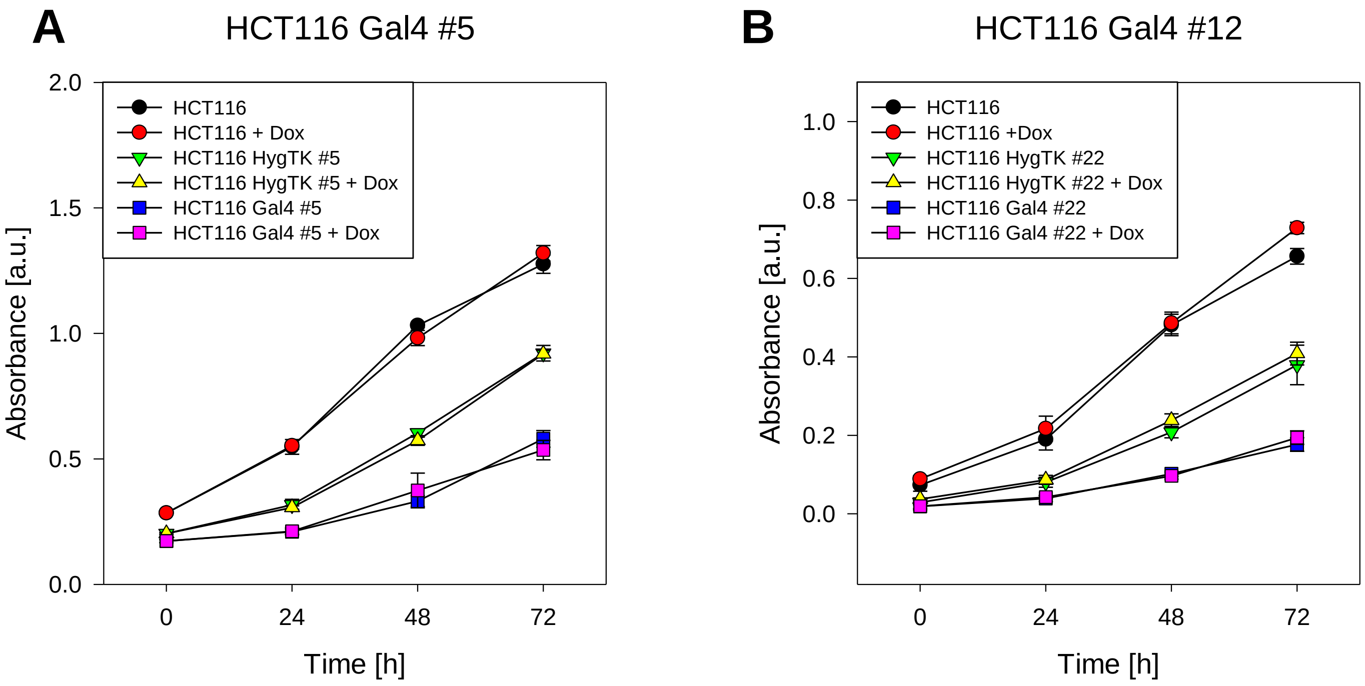

Figure S1. Effect of Doxycycline and Gal4 re-expression on cell proliferation. Model cell lines HCT116 Gal4 #5 (A) and #22 (B) as well as HCT116 and master cell lines HCT116 HygTK #5 and #22 were assayed in the presence or absence of doxycycline (0.5 µg/ml (A) and 0.75 µg/ml (B)) and data were obtained at the indicated time points. Measured absorbance represent relative cell numbers in the MTS assay. Results are the means of four independent series (+/-S.D.).
