# Supplementary material for: Combining Recombinase-Mediated Cassette Exchange Strategy with Quantitative Proteomic and Phosphoproteomic Analyses to Inspect Intracellular Functions of the Tumor Suppressor Galectin-4 in Colorectal Cancer Cells
Source: Int J Mol Sci. 2022 Jun 8;23(12):6414. doi: 10.3390/ijms23126414 (PMC9223697; doi:10.3390/ijms23126414)
Supplement: Supplementary file 1 [file ijms-23-06414-s001.zip › Figure S2.pdf]

Figure S2.

A

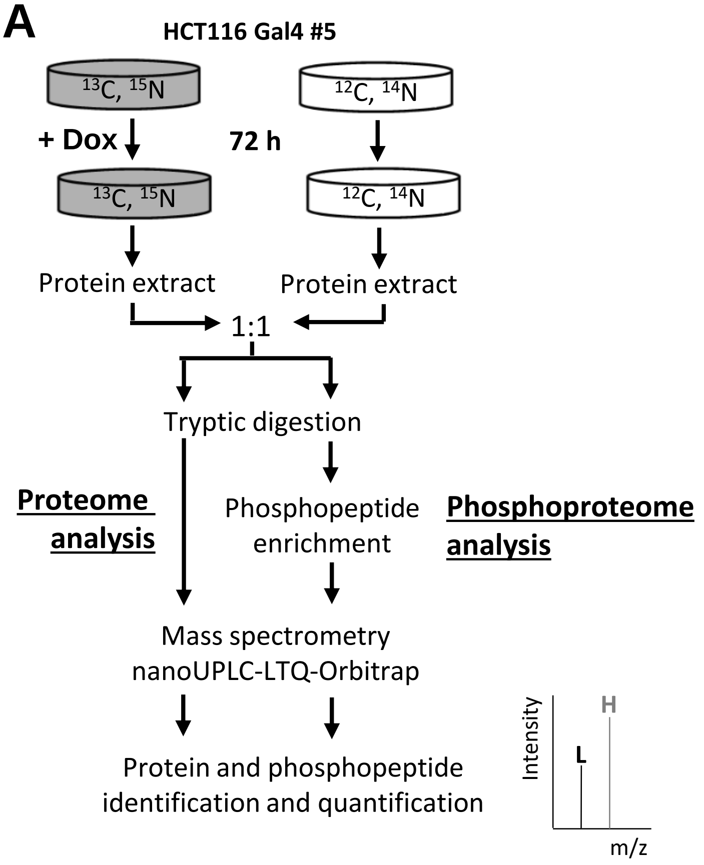

B

**Proteins**	**Total #**
Identified and quantified	3083
Regulated*	20
**Phosphosites**	
Identified and quantified	2071
Unknown§	32
Corrected for protein expression	1063
Regulated sites (phosphoproteins)	48 (21)

Figure S2. SILAC-based proteomic and phosphoproteomic analysis: (A) Workflow: SILAC labeling with Arg-10 and Lys-8 was applied to HCT116 Gal4 #5 cells followed by treatment with doxycycline (0.5 µg/ml) and mass spectrometric analysis leading to protein and phosphopeptide identificiation and quantification. (B) Summary of the proteome and phosphoproteome profiling upon Gal4 expression (*more than 1.5-fold in at least 2 biological replicates; §according to PhosphoSitePlus).
